# Sensory Feedback Training for Improvement of Finger Perception in Cerebral Palsy

**DOI:** 10.1155/2015/861617

**Published:** 2015-06-01

**Authors:** Tobias Blumenstein, Ana Alves-Pinto, Varvara Turova, Simon Aschmann, Ines Lützow, Renée Lampe

**Affiliations:** ^1^Research Unit for Cerebral Palsy and Children Neuroorthopaedics of the Buhl-Strohmaier Foundation, Department of Orthopedics, Clinic “Rechts der Isar”, Technical University of Munich, 81675 Munich, Germany; ^2^Center for Cerebral Palsy (ICP), 81377 Munich, Germany; ^3^Markus Würth Foundation Professorship of the Technical University of Munich, 81675 Munich, Germany

## Abstract

*Purpose*. To develop and to test a feedback training system for improvement of tactile perception and coordination of fingers in children and youth with cerebral palsy. *Methods*. The fingers of 7 probands with cerebral palsy of different types and severity were stimulated using small vibration motors integrated in the fingers of a hand glove. The vibration motors were connected through a microcontroller to a computer and to a response 5-button keyboard. By pressing an appropriate keyboard button, the proband must indicate in which finger the vibration was felt. The number of incorrect responses and the reaction time were measured for every finger. The perception and coordination of fingers were estimated before and after two-week training using both clinical tests and the measurements. *Results*. Proper functioning of the developed system in persons with cerebral palsy was confirmed. The tactile sensation of fingers was improved in five of seven subjects after two weeks of training. There was no clear tendency towards improvement of selective use of fingers. *Conclusion*. The designed feedback system could be used to train tactile perception of fingers in children and youth with cerebral palsy. An extensive study is required to confirm these findings.

## 1. Introduction

Cerebral palsy (CP) summarizes a group of disorders characterized by motor and sensory deficits caused by a nonprogressive damage of the brain in early phases of development [1]. The incidence of CP is about 2 per 1000 births [2, 3]. Between 30 and 50% of the patients with CP demonstrate sensory disturbances of the fingers [4, 5]. In children with spastic hemiplegia sensory deficits in hands are rather the rule than the exception [6]. But even individuals with CP who have mild motor deficits demonstrate ubiquitous tactile sensory impairments in upper limbs [7]. Tactile sensory impairments can lead to difficulties in grasping, in the selection of finger movements, and in the exploration of objects [8] as well as negatively affecting handwriting skills [9].

Since better tactile sensation supports motor learning [10], the recovery of tactile sensory function is very important rehabilitation task. However, a systematic review reported [11] an absence of intervention studies showing a reduction of tactile dysfunction in children with CP and confirmed a need for research on treatments aimed specifically at improving tactile sensory impairments in children with CP. Several tactile treatments like stimulus specific training [12], transfer enhanced training [12], and mirror therapy were regarded as deserved further investigation.

Other studies showed positive effect of sensory feedback systems in rehabilitation of patients with neurologic disorders [12–15]. Such systems are used, for example, to improve muscle control and balance to achieve a posture correction or make progress to motor function after a long time training phase [12]. Also advances in fine motor skills and coordination of finger movements while writing by using a haptic writing tablet were described in [13]. Using this method it was possible to significantly improve the writing ability of children with CP. According to our experience, biofeedback training like playing piano with digital interface can induce positive neuronal changes in children with CP [15]. An overview of methods of biofeedback therapy through visualization or auditory input for patients with a reduced physical condition is given in [16].

The purpose of this study was to develop a new feedback system aimed at reducing tactile sensory deficits of fingers and improving selective use of fingers in children and youth with CP. The training system delivers a harmless vibration to individual fingers of the hand to produce a tactile sensation and, in a playful manner, provide the patient with information about his response of the finger that was stimulated. Vibrations are detected by the mechanoreceptors in the skin and provide a time-variable stimulation to the skin that is easier to be detected [17]. The paper presents preliminary results on usability and potential efficacy of designed feedback system. Experiments were performed in children and youth with different causes and severity of CP.

## 2. Materials and Methods

### 2.1. System Design

**Figure 1:**
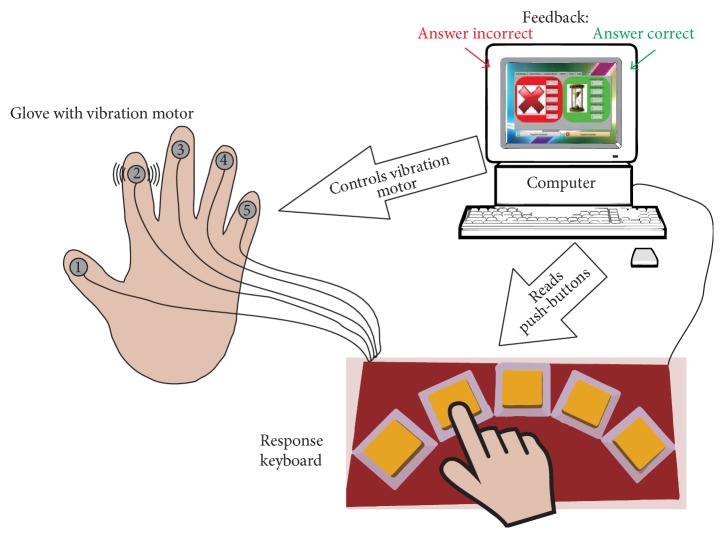
Schematic representation of the feedback game. The computer controls the vibration motors and reads the push-buttons.

Small vibration motors were attached to the individual fingers of a hand glove (Figure 1). The motors were controlled externally by a microcontroller and produced vibrations in each of the fingers. The microcontroller provided an interface among the vibration motors, buttons of a response keyboard, and a computer located underneath the buttons and connected to the microcontroller via USB. The response keyboard contained five buttons, each button, from the left to the right, associated with a different finger (the left button associated with the leftmost finger, etc.). The buttons were commercially available light switches and had a large enough size to facilitate the use by patients with restricted hand motor function. The keyboard and all its components had no sharp edges or corners that could cause injuries. The bottom side of the board had a nonslipping surface and the top side was completely washable for hygienic reasons. The vibration motors were attached to the glove fingers by hook-and-loop tape and were similar to that used in mobile phones. This made possible the use of different gloves (e.g., with different sizes) with the same keyboard and therefore speeded up the consecutive testing of several patients. To minimize the disturbance of finger movements, vibration motors were attached to the nail side of the gloves instead of finger pad side. The nail side is also preferable because of more direct transmission of vibration.

### 2.2. Software

The software was written in LabVIEW. The user interface was designed for easy use, especially for children and youth with disabilities. The software reads the button responses through the microcontroller, controls the vibration motors in the glove, and records the measurement data. The following data are stored: (1) which finger was stimulated, (2) which button was pressed, and (3) the time period from the start of the vibration to the response through the button press.

### 2.3. Sensorimotor Task

The sensorimotor task was developed as a feedback game. The participant wore a glove with built-in vibration motors (Figure 1). The motors delivered vibrations to each of the fingers separately, one at a time. The game consisted in indicating by means of pressing of a corresponding button on the keyboard which finger received the vibration. The subject received also a visual feedback on whether the response was correct or not. The subject's aim was to perform the task as accurate as possible (with least possible errors). A more detailed description of the experimental procedure used in the study is described below.

### 2.4. Experimental Procedure

**Figure 2:**
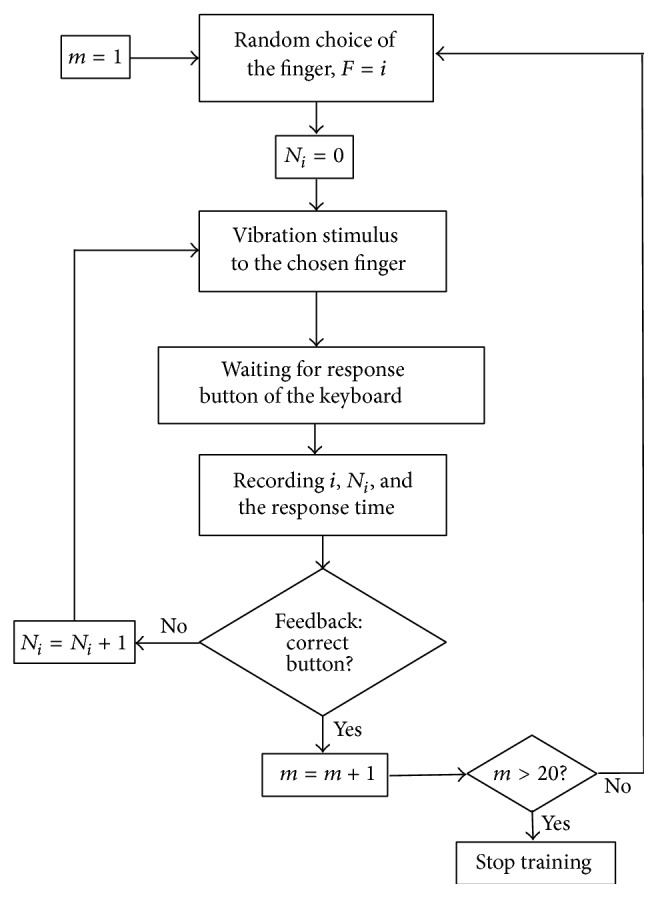
Training procedure (*m*: the number of trials, *i*: the finger number, and *N*_*i*_: the number of errors for the finger *i*).

The experimental procedure is explained using the flowchart in Figure 2.

All subjects received the same vibration amplitude and frequency (1 G at 200 Hz). The intensity of the vibration was defined by the technical characteristics of the vibration motors. Furthermore amplitude was such that all participants could easily perceive it and were not hurt by it. To exclude the influence of visual feedbacks, the participants were advised not to look at their hands during the session but at the therapist. The subjects were informed they should focus on pressing the right button and not do the task as fast as they could. Both hands were tested. Dominant hand was tested first, followed by nondominant hand. A minimum of 20 trials were conducted for each hand. Some participants, however, that performed the 20 trials in a short period of time requested to do more trials. In this case, an additional 10 trials (i.e., maximum 30 trials in total) were presented and responses were recorded. The fingers of each hand were stimulated in a random order, and the average number of trials per finger lies between 4,9 and 5,7. The duration of each treatment session including preparation was about 20 minutes per hand.

After completing the feedback game, the number of errors per finger and per training day was analyzed. Since different fingers were stimulated different times, the number of errors was divided by the number of trials per finger. The average response time, calculated across all performed trials, was also examined.

In total 8 training days were performed within 2 weeks. The first of the 8 training days was removed from the analysis, since the participants had to become familiar with the feedback device.

### 2.5. Sample

Four children and three youths with CP of different types and severity participated in the feedback training. Two persons were diagnosed with acquired CP after traumatic brain injury (TBI), two were diagnosed with left-sided unilateral spastic cerebral palsy (USCP), and three were diagnosed with bilateral spastic cerebral palsy (BSCP). Their clinical data are given in Table 1. The inclusion criteria were the presence of hand tactile sensory and motor deficits being identified using clinical tests described below. Those subjects who were not able to understand the task were excluded. All experimental procedures were approved by the Ethic Committee of the Faculty of Medicine. Informed consent was obtained from all participants, and when necessary from the parents, before starting the measurements.

During the study, none of the participants interrupted his/her general rehabilitation and training program at a center for persons with CP. The therapy program ran during school time and comprised speech, physio-, and occupational therapy.

## 3. Clinical Examination

Before the study all test subjects were classified according to the Gross Motor Function Classification System (GMFCS) [18] and the Manual Ability Classification System (MACS) [19]. Moreover, the following clinical investigations were performed: (a) determination of hand dominance, (b) presence of hand flexion contracture, (c) finger opposition test, and (d) proof of forearm supination ability. The GMFCS and MACS levels and results of the clinical examinations are given in Table 1.

Three tests were performed before and after the study:

1. tactile sensation of fingers,
2. fingers calling,
3. Box and Block test (see [20]).

The tactile sensation of fingers was tested in the following way (see e.g., [21]): each individual finger was brushed with a cotton swab and/or with a finger. The children had to identify and assign the respective finger during brushing of the finger. If a child was not able to call the perceived finger (due to cognition disturbance), he or she could point out to the finger felt. The test was carried out with closed eyes. Two trials with the same result for every finger provided clear evidence on the presence or absence of tactile sensation deficit.

In the “finger calling” test, the task was to point at those fingers that were called by the therapist. Whilst the previous test of tactile sensation assessed the proprioceptive abilities of the participant, the finger calling test was aimed at assessing to what extent the patient can identify and selectively move certain fingers, or, in other words, how accurate is the “internal body map” and, on the absence of external proprioceptive stimulation, the patient is able to move certain fingers. The decision was taken after a successful trial of maximally two trials.

In the Box and Block test, the task was to lift and move wooden cubes 2.5 × 2.5 × 2.5 cm in size over a 15-cm-high partition. As many as possible wooden cubes should be moved during a 1-minute time interval.

## 4. Results

All the test subjects were able to perform training with the designed system. The measurement results were successfully stored on the computer.

The results of the tests from 1 to 3, finger tactile sensation, fingers' calling, and Box and Block tests, are presented in Tables 2 to 4.

Both improvements in fingers tactile sensation and fingers recognition were observed.

The results of tactile sensation test are presented in Table 2. The fingers with impaired tactile perception before and after training are indicated. Before the training, 18 of 70 fingers (25,7%) were recognized as having abnormal sensitivity. After the training, only 11 (15,7%) such fingers remained, which means that 10% more fingers with intact tactile sensitivity were detected across all 7 subjects. This improvement was even more visible in fingers of nondominant hands (17,1% fewer fingers with impaired tactile sensation after training). In 5 subjects, the sensitivity of fingers was improved in nondominant hands (compare Tables 1 and 2). In two subjects with BSCP no objective improvement was observed, neither in fingers of dominant hand nor in fingers of nondominant hand (compare Tables 1 and 2).

The results of finger calling test are given in Table 3. Those fingers that were not correctly called are specified. The ability to recognize fingers was disturbed in 4 of 7 subjects. In two subjects, an improvement was detected, in one subject no changes were observed, and in one subject even a regression in recognition of the ring finger of both hands was established. Totally 20 fingers of 70 (28,6%) were called incorrectly before training. After training, the number of finger miscallings decreased to 12 fingers (17,1%). This result was even more pronounced in fingers of nondominant hands; the number of miscallings decreased from 31,4% to 14,3%.

In the Box and Block test, a Wilcoxon signed-rank test (WT) was used to determine whether the number of moved cubes is significantly different before and after the training. The significance was set at *p* < 0.05. According to obtained *p* values (see Table 4) the difference was not significant.

**Figure 3:**
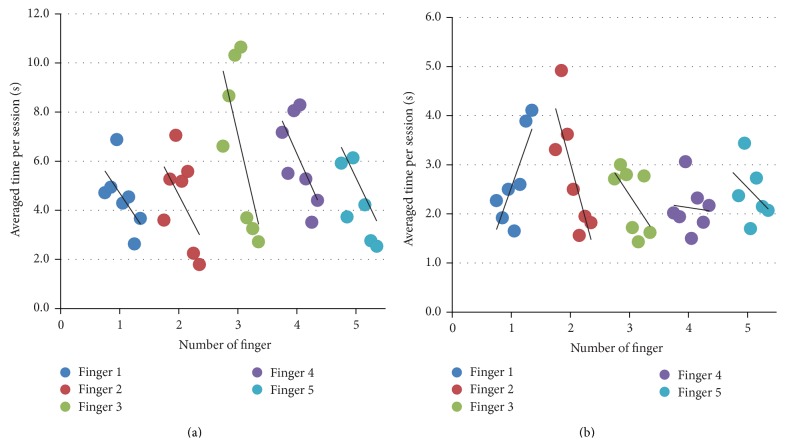
Exemplary analysis of the average times between the start of vibration in the finger and the response ((a) = nondominant hand and (b) = dominant hand). Each individual point describes a training day and the black line shows the fitted trend.

Of particular interest was whether the above described feedback training can improve the nondominant hand in the number of errors and in the response time. It turned out that the number of errors decreased in 16 of 35 fingers (45.7%) of nondominant hands, remained unchanged in 6 fingers (17.1%), and increased in 13 fingers (37.2%). Analysis of the number of errors in the dominant hands and the average response times in both hands with the Wilcoxon signed-rank test revealed no significant changes over the whole group. However, there were individual subjects for which a positive trend in the response time was established. Such an example is shown in Figure 3. Here, the average response time, that is, the time between the presentation of the vibration and the response by pressing a button on the keyboard, is presented for each finger of the subject. The leftmost data point for each finger corresponds to the first training day and the rightmost point corresponds to the last training day. The black lines show the fitted trend. A negative slope of the line means that the response time decreased in consecutive training days, implying a trend towards improvement in the recognition of fingers.

## 5. Discussion

Many mechanoreceptors which are located especially on the hands can perceive and differentiate the vibrations [17]. Hence it is reasonable to hypothesize that the vibration will be well perceived by its direct effect on the fingers skin and can help to improve tactile sensitivity and recognition of fingers. The experimental feedback training device was designed for improvement of tactile sensation and recognition of fingers in children and youth with finger sensitivity and coordination problems due to brain injuries.

Despite the short training period of 2 weeks, an improvement in the sensitivity and in the recognition of fingers was established. These results were more pronounced in nondominant hands, which is in accordance with the evidence that the nondominant hand has more potential for improvement. Also, the number of errors measured in fingers of nondominant hands was generally decreased. These findings are encouraging and support the potential benefit the training can have in children and youth with CP.

There was no significant difference in the before and after training scores in the Box and Block test. Also, the decrease in the average response time was not pronounced, although an effect was seen in individual cases (example subject in Figure 3). However, since motor and sensory functions cannot be considered separately from each other and the improvement in sensory abilities will likely contribute to the improvement of motor skills (see [22]), we expect that an improvement of motor skills and finger coordination can be achieved over a longer training period, which will result in the decrease of the response time and in better scores on the Box and Block test. A long term study is necessary to confirm this assumption. Moreover, a larger sample size is required to conduct meaningful statistical analysis.

The designed feedback system is suitable for children and youth with spastic muscle tone, coordination, and sensory deficits of upper extremities. All the participants of the study rated the feedback training as fun, easy to use, and beneficial for their hands.

Also the therapists reported about significant attention and the desire of all participants to continue the therapy. Due to the low cost and easy handling, the feedback training can also be done at home, which can additionally reduce the therapy costs.

## 6. Conclusions

A feedback system was developed for training of tactile sensitivity and selective use of fingers in children and youth with CP. Feasibility of this training method was demonstrated in a preliminary study in which 5 subjects with congenital CP and 2 subjects with acquired CP after TBI were trained over two weeks. Our findings provide preliminary evidence that the designed system can be easily implemented and the training with this system could be beneficial for improvement of tactile sensitivity and selective use of fingers. Additional studies are needed to quantify the extent of benefit and to compare our approach with traditional rehabilitation methods.

## Figures and Tables

**Table 1:** Patients' clinical data (r = right hand, l = left hand, HP = hemiparesis, USCP = unilateral spastic cerebral palsy, BSCP = bilateral cerebral palsy, TBI = traumatic brain injury, D1 = thumb, D2 = index finger, and D3 = middle finger).

	Patient number						
	1	2	3	4	5	6	7
Age (years)	12	17	15	17	10	18	11
Gender	f	m	f	f	m	m	m
Diagnosis	Right-sided HP after TBI	Right-sided HP after TBI	Left-sided USCP	Left-sided USCP	BSCP	BSCP	BSCP
GMFCS	III	III	II	II	III	III	II
MACS	r V l III	r V l III	r I l V	r I l V	III	II	II
Hand dominance	l	l	r	r	l	r	l
Hand flexion contracture	No	r	l	l	No	No	No
Opposition of fingers	r: D1–D3 possible l: norm	r: not possible l: norm	r: norm l: not possible	r: norm l: not possible	r: norm l: norm	r: norm l: norm	r: norm l: norm
Forearm supination	r: limited l: norm	r: not possible l: norm	r: norm l: not possible	r: norm l: limited	r: limited l: limited	r: norm l: norm	r: slightly limited l: slightly limited

**Table 2:** Tactile sensation of fingers before and after training. The fingers with abnormal sensitivity are indicated (D1: thumb, D2: index finger, D3: middle finger, D4: ring finger, and D5: little finger).

Patient number	Hand			
	Dominant		Nondominant	
	Time of exam			
	Before training	After training	Before training	After training
1	—	—	D4, D5	—
2	—	—	D3, D4, D5	—
3	—	—	D4, D5	D5
4	—	—	D1, D4	—
5	D4, D5	D4, D5	D3, D4	D4, D5
6	D3	D3	D4, D5	—
7	D3	D3, D4, D5	D3	D3, D4

**Table 3:** Results of the fingers' calling test. The fingers that were not correctly called are indicated (D1: thumb, D2: index finger, D3: middle finger, D4: ring finger, and D5: little finger).

Patient number	Hand			
	Dominant		Nondominant	
	Time of exam			
	Before training	After training	Before training	After training
1	D2–D5	—	D2–D5	—
2	—	—	—	—
3	—	—	—	—
4	—	—	—	—
5	D1–D5	D1–D5	D1–D5	D1, D2, D5
6	D3	D3	D3	D3
7	—	D4	—	D4

**Table 4:** Results of the Box and Block test. Number of moved wooden cubes before and after training and *p* values in Wilcoxon signed-rank test (WT) are given.

Patient number	Hand			
	Dominant		Nondominant	
	Time of exam			
	Before training	After training	Before training	After training
1	23	20	4	2
2	49	38	0	3
3	71	65	0	1
4	85	87	3	8
5	40	31	30	27
6	28	29	30	30
7	34	36	29	36
WT, *p* value	0.804		0.748	
